# Synthesis, Surface and Antimicrobial Activity of New Lactose-Based Surfactants

**DOI:** 10.3390/molecules24214010

**Published:** 2019-11-05

**Authors:** Katarzyna Michocka, Katarzyna Staszak, Daniela Gwiazdowska, Daria Wieczorek

**Affiliations:** 1 Department of Technology and Instrumental Analysis, Institute of Quality Science, Poznan University of Economics and Business, al. Niepodleglosci 10, 61-875 Poznan, Poland; daria.wieczorek@ue.poznan.pl; 2 Institute of Technology and Chemical Engineering, Poznan University of Technology, ul. Berdychowo 4, 60-965 Poznan, Poland; katarzyna.staszak@put.poznan.pl; 3 Department of Natural Science and Quality Assurance, Institute of Quality Science, Poznan University of Economics and Business, al. Niepodleglosci 10, 61-875 Poznan, Poland; daniela.gwiazdowska@ue.poznan.pl

**Keywords:** lactose-based surfactants, O-β-D-Galactopyranosyl-(1→4)-*N*-alkyl-(3-sulfopropyl)-D-glucosamine hydrochloride, CMC, surface tension, foamability, antimicrobial activity

## Abstract

This work presents a synthesis method for new surfactants based on lactose. The compounds obtained belong to the homologous series of O-β-D-Galactopyranosyl-(1→4)-*N*-alkyl-(3-sulfopropyl)-D-glucosamine hydrochloride, containing 12 and 14 carbon atoms in the alkyl chain, and they may serve as an example of cationic surfactants. The newly synthesized compounds exhibit good surface properties, low value of CMC (Critical Micelle Concentration) and good wetting properties. These surfactants’ ability to produce foam is considerably higher than in the commercial surfactants. Moreover, antibacterial and fungistatic activity was carried out by well diffusion assay against the selected bacteria (*Staphylococcus aureus*, *Bacillus subtilis*, *Escherichia coli* and *Pseudomonas aeruginosa*), yeasts (*Candida albicans*) and filamentous fungi (*Fusarium graminearum*, *F. avenaceum*, *F. oxysporum*, *F. culmorum*, *F. equiseti*, *Alternaria alternata* and *Botrytis cinerea*). It was shown that the resulting quaternary salts significantly inhibit the growth of tested microorganisms. Antibacterial and fungistatic activity of the surfactant compounds varied depending on the species of bacteria or fungi. The results of antimicrobial activity of new lactose derivatives indicate that the compounds exhibit larger or similar antagonistic activity against tested bacteria and fungi than typical cationic surfactant cetylpyridinium chloride.

## 1. Introduction

In the last few years there has been increased interest in work focused on synthesis and properties of new surfactants based on natural products. Literature present some examples of that type of surfactants based on sugars, sterols or fatty acids [1,2,3,4]. The reasons for choosing natural raw materials are: [5] renewable substrates (starting materials)—derived from continuous ecological cycles; [6] low cost of substrates—generally they are easy to obtain in nature, i.e. sucrose, lactose, glucose; [7] low toxicity and high biodegradability—the starting material come from nature therefore microorganisms adapted to their degradation can be found in the environment; [8] variety of products—surfactants have very broad range of structural diversities, i.e. sugars (monosacharides, disacharides, etc.) may be connected to the hydrophobic tail at any of their hydroxyl groups and by different types or connecting linkages. Moreover the hydrophilicity of sugar can be modified by reduction, oxidation, or addition of hydrophilic groups, such as a sulfonic acid residue or a polyoxyethylene chain. The hydrophobic moiety or moieties can also be changed by different reactions [4]. 

Taking into account the properties of surfactants derived from natural products they can successfully replace the typical surfactants based on petrochemical sources. Some of them are produced on a large scale by various producers, such as the glucose-based alkyl polyglucosides (APG) a non-ionic surfactant made from vegetable oils and starch. Germany-based Cognis, now owned by compatriot firm BASF, is the world’s largest APG surfactant producer. US-based Colonial Chemical has produced sugar surfactants made from corn syrup and coconut oil since 2002, under the brand Suga and PolySuga. France-based Wheat Oleo is the producer of alkyl polypentosides (APPs) surfactants made from combined natural fatty alcohols and pentose sugars coming from agricultural by-products wheat bran and straw [5]. Sorbitol is also widely used for preparing surfactants, notably as esters of fatty acids (tween, polysorbate) [6]. Formulations of the alkyl polyglycosides (APGs) are used in a variety of consumer products for laundry, hair and skin care [9]. In addition, sorbitan esters and sucrose esters are proposed in the food, cosmetic and pharmaceutical industries [10]. Surfactants based on sugars, obtained at laboratory scale but with very interesting properties, are widely described in the literature. For example, Imamura and co-workers tested sugar surfactants with different alkyl chain lengths and sugar head groups (sucrose C8-C16; glucose C8-C10, maltose C8) for their protein-stabilizing effect during freeze–thawing and freeze-drying. The investigation showed that sugar surfactants have the ability to stabilize a protein during freezing and freeze-drying [7]. The surfactants based on D-glucose, D-mannose, and D-galactose and triethylene glycolate spacer to provide additional water solubility were synthesized by Adam et al. and their photocontrollable ice recrystallization inhibition activity were presented [8]. Sakai et al. [11,12] studied the adsorption and micellization behavior of novel sugar gemini surfactants with glucono- and lactono- headgroups and compared to the corresponding monomeric surfactants. The investigation showed that greater efficiencies of the gemini surfactants in the adsorption and micellization result from the greater degree of molecular packing. Moreover the cationic/nonionic heterogemini surfactants containing a quaternary ammonium salt and gluconamide headgroups with different hydrocarbon chain lengths were synthesized and their adsorption and aggregation properties were investigated by the Yoshimura group [13].

The basic principle of sugar-based surfactants synthesis is fact that cyclic forms of sugars with a free reducing group react with ammonia, alkyl- and arylamines, esters of amino acids and urea derivatives giving the corresponding glycosylamines derivatives [14]. Thus the aim of this study was to use the main component of whey—lactose—to obtain new cationic surfactants and define their basic properties, such as: surface activity, wettability, foamability and antimicrobial activity.

## 2. Experiment

### 2.1. Materials

The new sugar-based surfactants, belonging to the homologous series of O-β-D-Galactopyranosyl-(1→4)-*N*-alkyl-(3-sulfopropyl)-D-glucosamine hydrochloride, containing 12 and 14 carbon atoms in the alkyl chain, were prepared by a multistep reaction. In the first step a series of N-alkyl-lactosamines were synthesized. Lactosamine derivatives were synthesized according to the method described in detail in patent literature [15] with the procedure presented in [16]. The appropriate n-alkylamine (0.1 M) reacted with lactose (0.1 M) in propan-2-ol as a solvent in room temperature. After solvent removal by evaporation, the reaction mixture was cooled at −10 °C and the crystal solids were obtained. The yields of reactions were 90% and 92% and melting temperatures 107 °C and 120 °C for N-dodecylaminelactosides and N-tetradecylaminelactosides, respectively.

**Figure 1:**
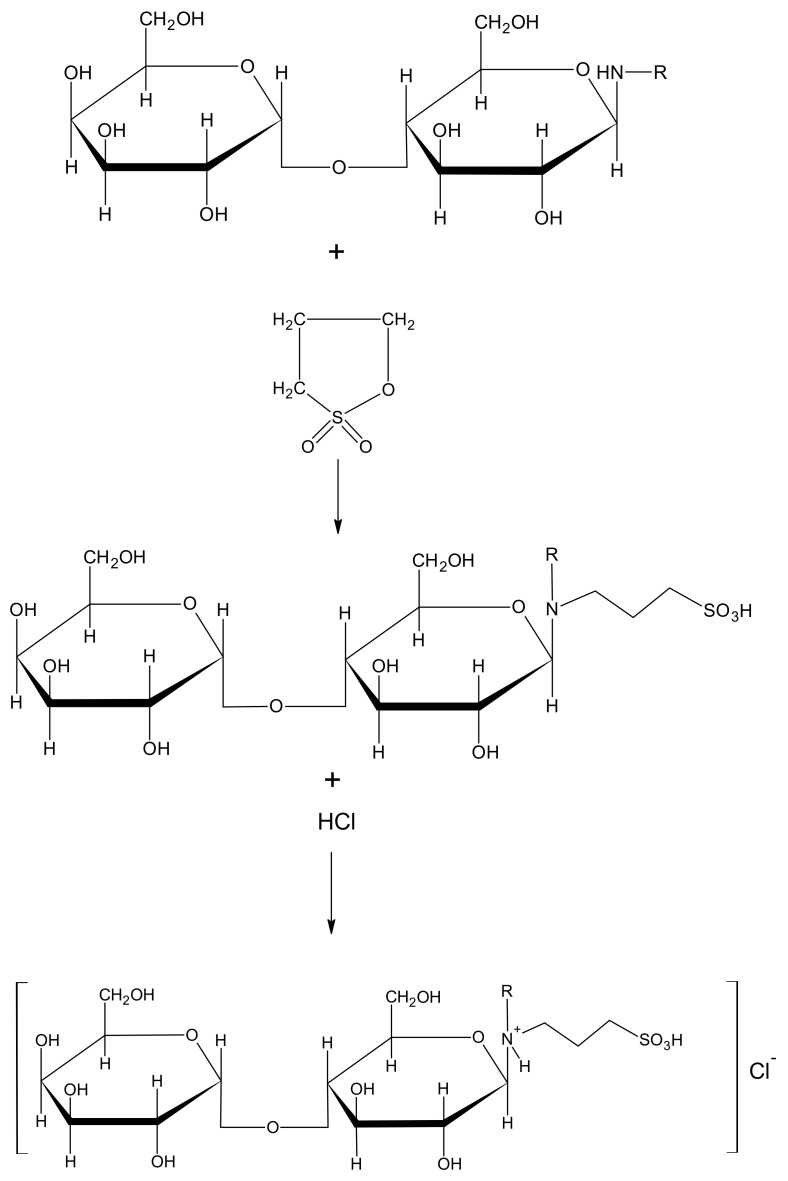
Synthesis procedure of O-β-D-Galactopyranosyl-(1→4)-*N*-alkyl-(3-sulfopropyl)-D-glucosamine hydrochloride.

In the next step, the 3-(N-alkyl-aminelactoside)-1-propanesulfonic acids were obtained by the reaction of equimolar amounts of the corresponding N-alkyl-lactosamines and 1,3-propanesultone with methanol as a solvent. The reaction schema is presented in first part of Figure 1. It was experimentally found that these compounds are insoluble in water and, therefore, in the next step their aqueous suspensions reacted with hydrochloric acid according to the method described in patent literature [17] to obtain the surfactants belonging to the homologous series O-β-D-Galactopyranosyl-(1→4)-*N*-alkyl-(3-sulfopropyl)-D-glucosamine hydrochloride. 

All reagents used in reactions were analytical grade. D-Lactose monohydrate P.P.H. POCh Gliwice; 1,3- Propanesultone, Fluka AG, Chemische Fabryk Büchs; propan-2-ol P.P.H. POCh Gliwice; Methyl alcohol Chempur, Piekary Śląskie; Dodecylamine Sigma-Aldrich Steinheim; Tetradecylamine Fluka AG, Chemische Fabrik Büchs.

### 2.2. Methods

#### 2.2.1. Synthesis Confirmation

The surfactants obtained along with intermediate reaction products were analyzed using spectroscopic and elemental analysis (Elementar Vario EL III) in order to confirm the structures of compounds obtained. Moreover, the structures were analyzed by means of nuclear magnetic resonance spectroscopy ^1^H NMR and ^13^C NMR (Varian Gemini 2000) at a frequency 300 Hz using deuterium oxide (D_2_O) as a solvent, and infrared spectra (Perkin Elmer Spectrum 100 with attenuated total reflectance (ATR) attachment).

#### 2.2.2. Surface Properties

The wettability and surface activities of surfactants’ aqueous solution were studied by means of equilibrium and dynamic surface tension measurements using drop shape method (Tracker IT Concept). Measurements were performed at a constant temperature of 297 K. The time of surface tension measurements depended on concentration of surfactants in the sample; the measurements were stopped once the equilibrium was reached. As suggested in [17], the equilibrium was established when a surface tension changed by less than 0.1 mN/m within a time period of 10 min. The measurements of surface tension was made in time interval about 0.3 s. Contact angle measurements were performed for aqueous solutions at a concentration 10 mM for 300 s. Surface used for measurements of contact angle were: aluminum, paraffin, PVC (poly(1-chloroethylene)), PTFE (poly(1,1,2,2-tetrafluoroethylene)), glass, PE (polyethene), PMMA (poly(methyl methacrylate). All the surfactant-based solutions were prepared with the use of water from Elga PURELAB Classic with resistivity = 18.2 MΩ·cm.

#### 2.2.3. Foaming Properties

The foaming properties of aqueous solutions of surfactants were set in a glass apparatus using the Ross–Miles method in accordance with the standard ASTM00A51E47 [18]. After creating column of foam, the foam height and volume were measured as a function of time. Foam volume recorded after 10 min was the basis for determining the stability of produced foam. The measurements of foaming properties were made on aqueous solutions for 6 different concentrations: 0.2, 0.4, 0.5, 0.6, 0.8 and 1%.

#### 2.2.4. Antimicrobial Activity

Antimicrobial activity of aqueous solutions of surfactants was determined by well diffusion assay against the selected microorganisms including bacteria, yeasts and molds. The group of bacteria consisted of Gram-positive bacteria: *Staphylococcus aureus* ATCC 33862 and *Bacillus subtilis* PCM 2021, as well as Gram-negative bacteria: *Escherichia coli* ATCC 8639 and *Pseudomonas aeruginosa* ATCC 10145. Antifungal activity was determined against yeasts *Candida albicans* ATCC 10123 and filamentous fungi *Fusarium graminearum* KZF 1, *F. avenaceum* KZF 3, *F. oxysporum* KZF 4, *F. culmorum* KZF 5, *F. equiseti* KZF 6, *Alternaria alternata* KZF 13 and *Botrytis cinerea* KZF 37. Filamentous fungi were obtained from the collection of the Department of Pesticide Investigation, Institute of Plant Protection–National Research Institute in Poznań, Poland. 

The bacterial and yeasts suspensions were prepared in sterile saline from 24 h cultures and standardized to obtain density 0.5 McFarland’s standard. Then, suspensions were overlaid with appropriate medium (nutrient agar Biocorp Sp. z o. o. Poland for bacteria, Sabouraud agar with chloramphenicol Biocorp Sp. z o. o. Poland for yeasts). The fungal suspensions were prepared in sterile saline with 50 µL of Tween-80 according to Kaur et al. [19]. The total spore count was determined using a haemocytometer and added to melted PDA (BioShop, Canada) to achieve a final spore count of 10^6^ spores mL^−1^. The mixture was poured in Petri plates.

After medium solidification, the wells (10 mm in diameter) were cut with a sterile cork borer to which 100 µL of surfactants solutions were introduced. The plates were incubated in the temperature of 37 °C for 24 h (bacteria and yeasts) and at temperature 20 °C for 5–7 days, depending on fungal species. After incubation, the diameters of inhibition zones were measured in millimeters. All the surfactant solutions were prepared using water from the PURELAB Classic, Elga with resistivity = 18.2 MΩ·cm, at the concentration of 1.0 and 0.5 g·cm^−3^. Tests were performed in triplicate and the mean values are presented.

### 2.3. Results and Discussion

#### 2.3.1. Synthesis Results

Both the structures of intermediates as well as obtained surfactants were confirmed by spectroscopic methods and elemental analysis. The results for surfactants are presented below.

*O-β-D-Galactopyranosyl-(1→4)-N-dodecyl-(3-sulfopropyl)-D-glucosamine hydrochloride (C12S3L)*

^1^H NMR (300 MHz, D_2_O): δ = 0.84 (t, 3H, CH_3_), 1.23-1.27 (m, 20H, (CH_2_)_10_), 1.65 (m, 2H, CH_2_), 1.96 (m, 2H, CH_2_), 2.20 (m, 1H, NH^+^) 2.89-2.96 (m, 4H, -CH_2_N, -CH_2_SO_3_), 3.33-3.92 (m, 21H, CH_2_,COH), 4.43 (m, 1H, SOH)

^13^C NMR (300 MHz, D_2_O): δ = 16.0 (CH_3_), 24.9, 26.6, 28.8, 29.3, 31.6, 31.9, 32.1, 32.2, 32.3, 34.3 (10 x CH_2_), 41.8 (CH_2_SO_3_), 50.3 (CH_2_), 62.5, 63.3, 70.8, 72.4, 73.0, 73.7, 74.8, 76.1, 76.6, 77.6, 80.7, 94.1, 98.0, 105.2 (CH_2_-lactose)

Infrared (IR) (KBr): 3450, 2910, 1710, 1480, 1220, 1050, 890, 790, 720, 610, 520 cm^−1^

Anal. Calcd: C, 42.46; H, 7.91; N, 1.05; S, 3.68. Found: C, 42.21; H, 7.91; N, 1.02; S, 3.03.

Mp. 118 °C, Yield of final reaction 94%. 

*O-β-D-Galactopyranosyl-(1→4)-N-tetradecyl-(3-sulfopropyl)-D-glucosamine hydrochloride (C14S3L)*

^1^H NMR (300 MHz, D_2_O): δ = 0.86 (t, 3H, CH_3_), 1.21-1.27 (m, 20H, (CH_2_)_10_), 1.69 (m, 2H, CH_2_), 1.98 (m, 2H, CH_2_), 2.18 (m, 1H, NH^+^) 2.90-2.96 (m, 4H, -CH_2_N_, -_CH_2_SO_3_), 3.32–3.92 (m, 21H, CH_2_,COH), 4.43 (m, 1H, SOH)

^13^C NMR (300 MHz, D_2_O): δ = 17.9 (CH_3_), 23.6, 24.2, 24.9, 26.1, 26.9, 29.0, 29.6, 31.9, 32.1, 32.2, 32.3, 34.3 (12 × CH_2_), 41.8 (CH_2_SO_3_), 50.3 (CH_2_), 62.5, 63.3, 70.8, 72.4, 73.0, 73.7, 74.8, 76.1, 76.6, 77.6, 80.7, 94.3, 98.6, 105.1 (CH_2_-lactose) 

IR (KBr): 3440, 2920, 1710, 1480, 1220, 1050, 890, 790, 720, 610, 510 cm^−1^

Anal. Calcd: C, 43.91; H, 8.15; N, 0.96; S, 3.49. Found: C, 43.39; H, 7.84; N, 1.20; S, 3.31.

mp. 121 °C, Yield of final reaction 95%. 

##### Surface Activity

**Figure 2:**
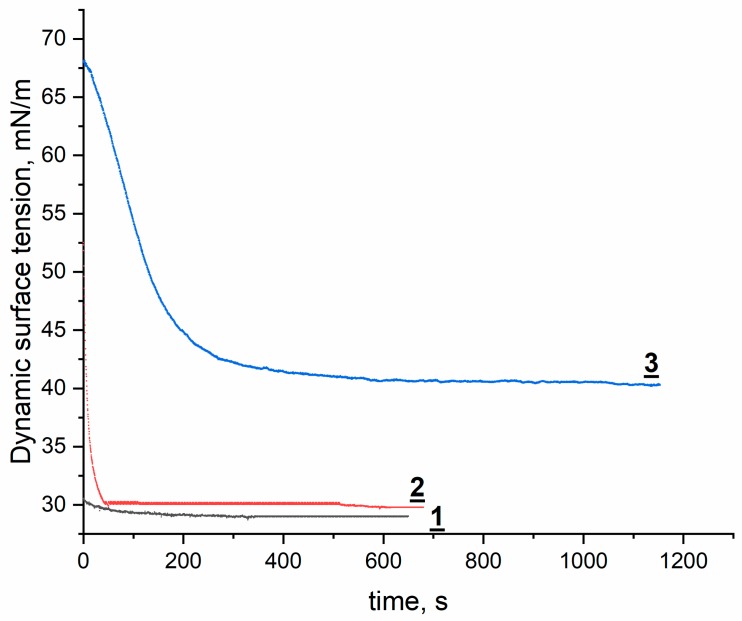
Dynamic surface tension for O-β-D-Galactopyranosyl-(1→4)-*N*-tetradecyl-(3-sulfopropyl)-D-glucosamine hydrochloride in concentrations: 1–30 mM, 2–0.3 mM, 3–0.0003 mM.

The adsorption process of surfactants is a dynamic one. For surfactants with low surface activity, the adsorption reaches its equilibrium state within seconds up to hours [16]. As was mentioned above, in this study, the equilibrium was established when a surface tension changed by less than 0.1 mN/m within a period of 10 min during the measurement as suggested in [17], which is presented in Figure 2 for various surfactant’s concentration. The curves are of typical shape with the dynamic and equilibrium zones with surface tension decreasing in time. This effect could be particularly observed in surfactant concentration which is higher but remains below CMC (Critical Micelle Concentration). The increase in the surfactant concentration gives way to the decrease of equilibrium surface tension, while the dynamic surface tension approaches the equilibrium value more rapidly. In higher surfactant concentrations, the dynamic surface tensions are decreased due to a significant initial surfactant load caused by adsorption during the dead time stage. This dependence was observed for both surfactants studied. Moreover, the same relationship was observed by other authors in other types of surfactants at the air/water interface i.e. sulfobetaines with N-alkylmorpholinium moiety [17], lactobionamide-type-sugar-based gemini surfactants [20], lipophilic silicone surfactants [21], and non-ionic polyoxyethylene alcohols [22,23].

**Figure 3:**
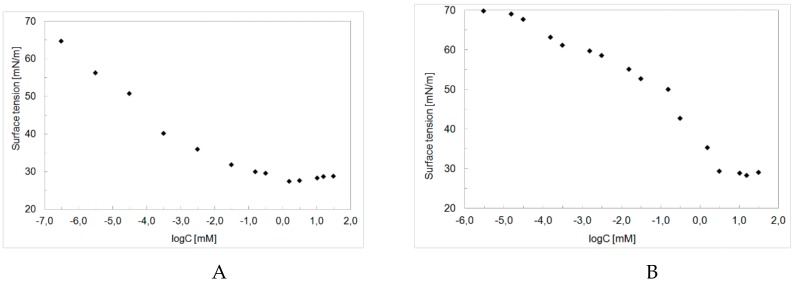
The surface tension isotherms for aqueous solutions of (**A**)–O-β-D-Galactopyranosyl-(1→4)-*N*-dodecyl-(3-sulfopropyl)-D-glucosamine hydrochloride, (**B**)–O-β-D-Galactopyranosyl-(1→4)-*N*-tetradecyl-(3-sulfopropyl)-D-glucosamine hydrochloride.

Figure 3 presents adsorption isotherms received for lactose-based surfactant derivatives with 12 and 14 carbon atoms in the alkyl chain. The more carbons in the alkyl chain, the greater the ability to reduce the surface tension, with the CMC value being shifted toward lower surfactant concentrations. The same relationship was observed by other authors. In work [24], the CMC of quaternary ammonium compounds (monoalkonium, dialkonium, and benzalkonium chloride groups) were presented. CMC values ranged from 230 to 5576 mg/L. The lowest CMC in each quaternary ammonium compound group was for those with the longest alkyl chain. Kopecky [25] concludes that in homologous series of quaternary ammonium compounds, log CMC decreases linearly with the number of -CH_2_ groups in the alkyl tail of the quaternary cations, so that with some limitations, log CMC is a relative measure of hydrophobicity. The effect described therein and works cited is due to increased hydrophobicity of the tail length, which significantly affects their CMC. The same impact of the alkyl chain length on the CMC value was observed also for other sugar-based surfactants such as urinate and uronoamide derivatives of dodecyl glucuronolactones [26], gluconamide-type surfactants [12,13] and sucrose monoesters [6].

The critical micellar concentration is not only a measure of solubility for surfactants but also a criterion which determines the effective application of concentration for disinfection. The effectiveness of quaternary ammonium compounds (an example of cationic surfactants) is the highest at CMC value [24]. It is connected with the amount of monomer present in the solution. The available concentration of the free, non-micellized surfactant ions in the aqueous solution is limited not so much by the solubility of the surfactant, but primarily by the value of CMC. The maximum possible concentration of the surfactant monomer is more or less equal to CMC value. Above CMC, the surfactant monomer concentration starts decreasing, while the total surfactant concentration in the micellar solution begins to increase. Thus, the concentration of the free monomer of surfactant increases up to CMC only, while in the micellar solution above CMC it sharply decreases in conformity with the mass-action micellization model [25]. CMC thus represents the maximal available concentration of the monomer of surfactant, which may cause the decrease of antimicrobial activity above CMC. 

Additional physicochemical analyses on the basis of the surface tension data were carried out using the Szyszkowski equation [27]:

(1) $$\gamma^{Sz}=\gamma_{0}\cdot \left[1-B_{Sz}\cdot ln\left(\frac{c}{A_{Sz}}+1\right)\right]$$

where *A_Sz_* and *B_Sz_* are the coefficients of Szyszkowski equation.

The values of surface excess at the saturated interface (Γ^∞^), the minimum molecular area in the adsorption layer at the saturated interface (*A_min_*), and the Gibbs free energy of adsorption (Δ*G_ads_*) for the systems in question were calculated according to the Equations [28,29]:

(2) $$\Gamma_{\infty }=\frac{B_{Sz}\gamma_{0}}{RT}$$

(3) $$A_{min}=\frac{1}{\Gamma_{\infty }N_{A}}$$

(4) $$\Delta G_{ads}^{Sz}=RTln\left(A_{min}\right)$$

where *γ*_0_, *N_A_*, *R*, *T* stand for the interfacial tension at concentration *c* = 0 (71.2mN/m), the Avogadro constant (6.022·10^23^ mol^−1^), gas constant (8.314 J/mol·K) and temperature (294 K), respectively. 

The e co values of adsorption parameters are presented in Table 1, wherein the coefficients B_sz_ were calculated from Equation (1) and were equal to 0.046 and 0.034 for C12S3L and C14S3L, respectively.

The surface excess at the saturated interface indicates the effectiveness of surfactant adsorption. It is closely related to the packing of surfactant molecules in the adsorbed film. The packing of adsorbed film at the air–water interface is an important factor in surfactant application such as foaming, wetting, detergency and emulsification. The values of Γ^∞^ decrease, with an increasing number of carbons in the hydrophobic tail. The same relationship was observed in a homologies series of other cationic surfactants—2-(alkyloxy)-N,N,N-tris (2-hydroxyethyl)-2-oxoethanaminium chloride [30].

The values of *A_min_* increase with increasing number of carbons in the hydrophobic tail, suggesting that surfactants with shorter hydrophobic tails have higher packing densities at the air–water surface. This phenomenon could be explained by longer hydrophobic chains being more prone to curl [31]. The values of Δ*G_ads_* are negative, indicating that the surfactants have a great ability to adsorb at the air–water interface, higher in the case of surfactant with a longer alkyl chain.

**Figure 4:**
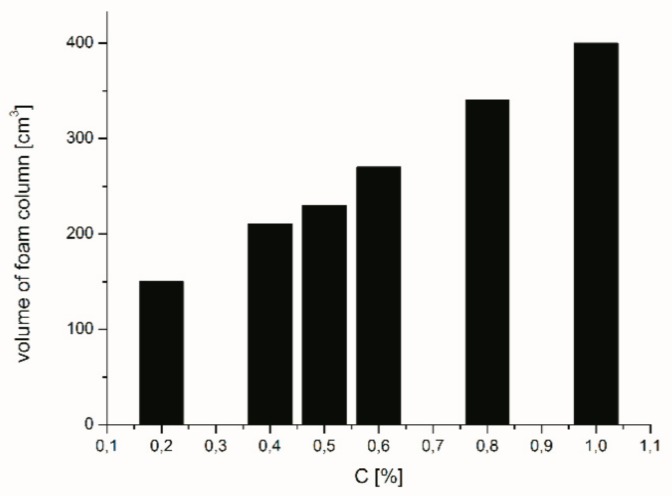
The effect of O-β-D-Galactopyranosyl-(1→4)-*N*-dodecyl-(3-sulfopropyl)-D-glucosamine hydrochloride concentration on volume of foam column.

In order to verify the usage properties of new cationic surfactants derived on the basis of lactose, the ability to produce foam was also tested. A study of foaming properties of aqueous solutions of the obtained compounds was performed using a modified Ross–Miles method [18]. These measurements were carried out for six different concentrations of aqueous solutions of O-β-D-Galactopyranosyl-(1→4)-*N*-alkyl-(3-sulfopropyl)-D-glucosamine hydrochloride. Figure 4 shows the dependence between the volume of foam column and concentration of surfactant in aqueous solution. As it is apparent from experimental and expected research, the volume of column foam increases along with surfactant concentration’s increase. 

Comparison of the foamability of aqueous solutions of surfactants obtained with aqueous solutions of commercial soaps shows that solutions of lactose-based surfactants produce significantly more foam than the soap solutions. For instance, 2% solution of liquid soap or cocamidopropylbetaine produce about 290 cm^3^ foam [32] and 350 cm^3^ [33] respectively, while 1% solution of O-β-D-Galactopyranosyl-(1→4)-*N*-dodecyl-(3-sulfopropyl)-D-glucosamine hydrochloride about 400 cm^3^. It is also very important to note that aqueous solutions of the surfactants at hand not only produce a very high column of foam but also exhibit high foam stability indicators. 

**Figure 5:**
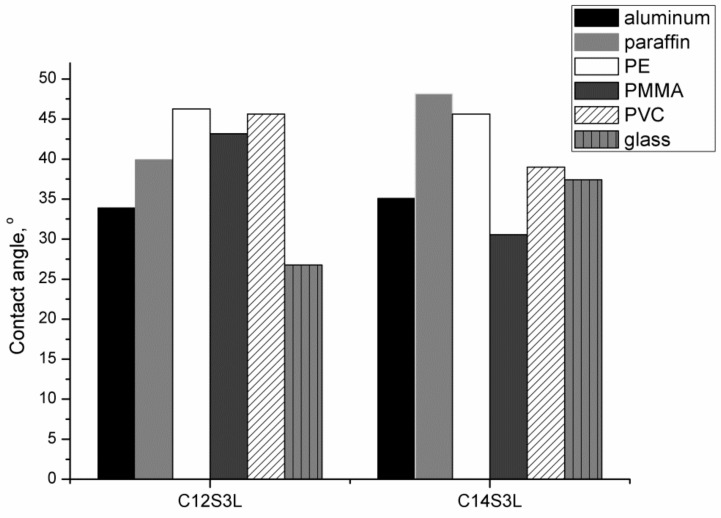
Wetting angles of different surfaces of aqueous solutions of O-β-D-Galactopyranosyl-(1→4)-*N*-dodecyl-(3-sulfopropyl)-D-glucosamine hydrochloride (C12S3L) and O-β-D-Galactopyranosyl-(1→4)-*N*-tetradecyl-(3-sulfopropyl)-D-glucosamine hydrochloride (C14S3L).

Many processes depend on wetting including those used in essential industrial processes as well. These processes include flotation, detergency, cosmetic, washing, lubrication, coating, deposition or enhanced oil recovery. Understanding and characterizing the wettability of solid surfaces through surfactants is thus of paramount importance for both theoretical study and practical application. Due to their bacteriostatic properties, cationic surfactants are widely used in cosmetic products and have contact with the skin surface. As it is very difficult to study surfactant adsorption and wetting properties on the human skin, polymer surfaces are often used as skin substitutes. As the human skin surface is classified as a low energy hydrophobic surface [34], the PMMA (poly(methyl methacrylate)) is proposed in such a study. PMMA surface is similar to the human skin due to similar γc value and the surface free energy [35]. Beside PMMA is a medium energetic polymer solid and surfactant molecules can also adsorb via hydrophobic interaction [36] Figure 5 presents values of wetting angles measured for different surfaces. The measurements were conducted for aqueous solutions of synthesized compounds at the concentration of 10 mM. The contact angle was determined for the following surfaces: glass, aluminum, paraffin, PMMA, PVC, PE. In general, cationic surfactants have a positive charge, and thus they adsorb strongly onto most solid surfaces (which tend to be negatively charged) [37]. The results indicate that the obtained surfactants have good wetting properties in the surface studied. Hence, these results confirm the general trend of wettability for such surfactants. The value of contact angle depends on concentration and it is difficult to directly compare results with literature data. However, it can be assumed that the value of contact angle for surfactant solution above CMC is rather constant [38]. Accordingly, investigations were performed at a concentration above the CMC and, with such values, they were compared with results obtained by other authors. For example, the values of contact angle for typical cationic surfactants: dodecylethyldimethylammonium bromide and benzyldimethyldodecylammonium bromide in the PMMA surface are equal to 42° and 27°, respectively [35], while for hexadecyl(trimethyl)ammonium bromide (CTAB) decrease from 82.7° to 51° at the CMC [39]. These values are very similar to those obtained for lactose-based surfactants. It is difficult to find correlation between the surfactants structure and considered solid surface. The same conculsion were presented in Sritapunya et al. [39]. The authours studied the contact angle for three anionic and three cationic surfactants on eight different polymers with varying hydrophobicity and concluded that contact angle for different surfactants are very similar, i.e. contact angle for CTAB, TTAB (tetradecyl(trimethyl)ammoniumbromide), DTAB (dodecyl(trimethyl)ammoniumbromide) and SDS for high density polyethylen varies form 33.4° to 36.4° at the CMC. Morover, as was presented in Lv et al. work [36] also in the case of xylyl-substituted biquaternary ammonium salt Gemini surfactants with different sapace (C3 and C6) there are no relationship between contact angle for PMMA and polytetrafluoroethylene (PTFE) and the kind of surfactants considered.

## 3. Antimicrobial Activity

The physical and chemical properties of quaternary ammonium compounds strongly affect not only their fate but also their toxicity, bioavailability and biodegradability. Quaternary ammonium compounds with relatively long alkyl chains (>=14 carbons) are more effectively eliminated during wastewater treatment by adsorption to biomass or by adsorption to organic and inorganic solids once they are released to the environment. As a result, quaternary ammonium compounds with short alkyl chains (<14 carbons) are more mobile and bioavailable than quaternary ammonium compounds with longer alkyl chains. Therefore, exposure of microorganisms to quaternary ammonium compounds with shorter alkyl chains is more probable [40,41].

Table 2 shows the results of a preliminary screening study which illustrate the interaction of investigated surfactants with different microorganisms, including Gram-positive and Gram-negative bacteria, yeasts and filamentous fungi selected from the genera *Fusarium*, *Alternaria* and *Botrytis*. Antimicrobial activity of tested compounds was differentiated and appeared to be dependent on both surfactant structure and indicator microorganism. However, surfactants inhibited the growth of all examined bacteria and fungi. Gram-positive bacteria and yeasts were more susceptible to O-β-D-Galactopyranosyl-(1→4)-N-dodecyl-(3-sulfopropyl)-D-glucosamine hydrochloride, while the sensitivity of Gram-negative bacteria to both surfactants was comparable.

Antifungal activity against filamentous fungi was more differentiated. It is worth noting that in some cases a completely clear zone around the well with tested compound was observed, while sometimes the zone of inhibition was connected with a distinct change in the structure and degree of mycelium. 

O-β-D-Galactopyranosyl-(1→4)-*N*-dodecyl-(3-sulfopropyl)-D-glucosamine hydrochloride showed the greatest antagonistic effect to *F. graminearum*. Growth inhibition zone was equal to 30 mm. Moreover, this compound limited the growth of *F. oxysporum*, *F. equiseti*, *F. culmorum* and *F. avenaceum*. No activity against *A. alternata* and *B. cinerea* has been observed. 

O-β-D-Galactopyranosyl-(1→4)-*N*-tetredecyl-(3-sulfopropyl)-D-glucosamine hydrochloride showed the highest antagonist activity against *F. graminearum* and slightly less against *B. cinerea* and *F. culmorum*. This surfactant also showed antifungal activity towards *F. avenaceum*, *F. oxysporum* and *A. alternata*. Nevertheless, in the case of *F. equiseti* O-β-D- Galactopyranosyl-(1→4)-*N*-tetradecyl-(3-sulfopropyl)-D-glucosamine, only hydrochloride restricted their growth.

It can be concluded that a stronger antagonistic effect against filamentous fungi was manifested in a surfactant with 14 carbon atoms in the alkyl chain (3 mm). A comparison of antimicrobial activity of obtained surfactants with activity of commercial cetyltrimethyloammonium bromide (CTAB) and cetylpyridinium chloride (CPC) indicates higher or comparable activity of tested compounds [42,43]. 

Comparative studies indicated that lactose based surfactants showed a stronger bactericidal effect towards all tested bacteria than commercial compounds. However, it should be noted that the potency of fungicidal action against *Alternaria alternata* and *Fusarium equiseti* is lower in comparison to the commercial well-known surfactants, or very similar to activity against *Fusarium avenaceum*, *Fusarium oxysporum* and *Fusarium culmorum*.

Although the literature confirms antimicrobial properties of cationic surfactants with sugar moiety, the information about antifungal activity towards filamentous fungi is pretty scarce. In addition, the authors underline that activity against microorganisms is dependent on structure, including the nature of carbohydrate moiety or hydrocarbon chain length, as was observed in papers referred to in this article [44], synthesized two sugar-based surfactants, lactose palmitoleate and lactose nervonate, which exhibited antimicrobial activity against eight pathogenic species belonging to Gram-positive species such as *Listeria monocytogenes*, *Enterococcus faecalis*, *S. aureus*, Gram-negative microorganisms including *E. coli*, *P. aeruginosa*, *Salmonella enteritidis* and *Yersinia enterocolitica* as well as against yeasts *C. albicans*. Except for *S. enteritidis*, which was more susceptible to lactose nervonate, other tested bacteria and fungi demonstrated similar sensitivity to both surfactants. A series of amphiphilic methyl glucopyranoside ethers synthesized by [45] demonstrated antimicrobial activity against Gram-positive bacteria such as *Listeria monocytogenes*, *Enterococcus faecalis*, *E. faecium* and *S. aureus*, including multi-resistant strains such as vancomycin-, methicillin- and daptomycin-resistant strains. It was also found that antimicrobial activity was related to the physicochemical properties of examined compounds. Also, other authors emphasise the relationship between structure and antimicrobial properties of sugar-based surfactants examined for surface activity, adsorption or aggregation behavior, as well as the antimicrobial activity of three saccharide-amide cationic surfactants varying in hydrocarbon chain length (10, 12 and 14 carbon atoms) [46]. The results showed that tested surfactant with an alkyl chain length of 12 carbon atoms demonstrated the highest efficiency, while antimicrobial activity of surfactant containing 10 carbon atoms was the worst. Similar observations were made by [47], who synthesized polysaccharide carbohydrate derivatives of sodium alginate surfactants and their complexes with cobalt, copper and zinc. Tested compounds were active towards Gram-positive and Gram-negative bacteria as well as fungi (*C. albicans* and *Aspergillus niger*). The authors stated that antimicrobial activity depended on the chemical structure of molecules and length of hydrophobic chain with the highest activity demonstrated by surfactant containing 12 carbon atoms. These results are in agreement with some other reports [48,49,50]. On the basis of research results, it can be concluded that the quaternary salts obtained, where the hydrophilic part is lactose, exhibit fungistatic activity against some filamentous fungi.

## 4. Conclusions

Experiments presented in this study lead to the conclusion that the lactose is a sugar which can be used as a substrate in the reaction of surfactants’ preparation. The evaluation of obtained surfactants’ surface properties indicated that these compounds have low CMC values and can significantly reduce surface tension. Moreover, new compounds have good wetting properties. Evaluation of foamability showed that lactose-based surfactants can be used as good foaming agents. The resulting surfactants exhibit not only excellent foaming ability but also the durability of produced foam. Results of antimicrobial activity of new lactose derivatives show that compounds exhibit larger or similar antagonistic activity against tested bacteria and fungi than typical surfactant-cetylpyridinium chloride.

The focus of attention in our studies falls on systematic comparison of petroleum-based surfactants and sugar-based surfactants with the aim of highlighting the benefits of sugar-based surfactant platforms.

## Figures and Tables

**Table 1:** Surface activity of O-β-D-Galactopyranosyl-(1→4)-*N*-alkyl-(3-sulfopropyl)-D-glucosamine hydrochloride.

Surfactant	CMC [mM]	γ_CMC_ [mN/m]	Γ^∞^·10^6^ [mol/m^2^]	Δ*G_ads_* [kJ/mol]	*A_min_*·10^18^ [m^2^]
C12S3L	3.01	29.5	1.34	−41.2	1.24
C14S3L	0.4	27.6	0.988	−64.2	1.68

**Table 2:** The growth inhibition [mm] of tested microorganisms by O-β-D-Galactopyranosyl-(1→4)-*N*-alkyl-(3-sulfopropyl)-D-glucosamine hydrochloride [zone inhibition in mm].

Microorganism Fungi	O-β-D-Galactopyranosyl-(1→4)-*N*-dodecyl-(3-sulfopropyl)-D-glucosamine hydrochloride	O-β-D-Galactopyranosyl-(1→4)-*N*-tetradecyl-(3-sulfopropyl)-D-glucosamine hydrochloride	Cetyl-pyridinium chloride	Cetyl-trimethyl-ammonium bromide
Gram-positive bacteria				
*Staphylococcus aureus*	20.8 ± 0.00	17.3 ± 1.15	12.0 ± 0	13.0 ± 0
*Bacillus subtilis*	26.6 ± 1.15	14.0 ± 1.15	12.0 ± 0	12.0 ± 0
Gram-negative bacteria				
*Escherichia coli*	18.6 ± 1.15	18.6 ± 0.00	11.7 ± 0	11.7 ± 0
*Pseudomonas aeruginosa*	16.0 ± 0.00	16.0 ± 0.00	14.0 ± 0	14.5 ± 0
Yeasts				
*Candida albicans*	20.0 ± 0.00	15.3 ± 1.15	14.67 ± 0	14.00 ± 0
Filamentous fungi				
*Fusarium graminearum*	28.0 ± 2.82	24.0 ± 2.82	13.5 ± 0	14.5 ± 0
*Fusarium avenaceum*	12.0 ± 0.00	19.0 ± 1.41	13.5 ± 0	16.5 ± 0
*Fusarium oxysporum*	12.0 ± 0.00	16.0 ± 0.00	12.0 ± 0	13.0 ± 0
*Fusarium culmorum*	12.0 ± 0.00	15.0 ± 1.41	13.5 ± 0	14.5 ± 0
*Fusarium equiseti*	12.0 ± 0.00	12.0 ± 0.00	13.5 ± 0	13.0 ± 0
*Alternaria alternata*	0.0 ± 0.00	16.0 ± 0.00	19.0 ± 0	19.0 ± 0
*Botrytis cinerea*	0.0 ± 0.00	20.0 ± 0.00	15.0 ± 0	16.0 ± 0
